# Cerebral X-linked Adrenoleukodystrophy Presenting As Enlarging Cavum Vergae Cyst: A Case Report

**DOI:** 10.7759/cureus.39353

**Published:** 2023-05-22

**Authors:** Tien Meng Cheong, Wan Tew Seow, Ronald Ming Ren Tan, Terrence Thomas, Si Min Chiow, Jeanette Goh, Syeda K Qadri, Sharon YY Low

**Affiliations:** 1 Neurosurgery, KK Women's and Children's Hospital, Singapore, SGP; 2 Neurosurgery, National Neuroscience Institute, Singapore, SGP; 3 Emergency Medicine, KK Women's and Children's Hospital, Singapore, SGP; 4 Pediatric Neurology, KK Women's and Children's Hospital, Singapore, SGP; 5 Radiology, KK Women's and Children's Hospital, Singapore, SGP; 6 Pediatrics, KK Women's and Children's Hospital, Singapore, SGP; 7 Neurosurgical Service, KK Women's and Children's Hospital, Singapore, SGP

**Keywords:** adrenoleukodystrophies, cerebral x-linked adrenoleukodystrophy, x-linked adrenoleukodystrophy, obstructive hydrocephalus, cavum vergae cyst

## Abstract

The cavum vergae cyst (CVC) is an uncommon brain malformation. Most patients with CVC are asymptomatic and do not require neurosurgical intervention. Separately, cerebral X-linked adrenoleukodystrophy (X-ALD) is one of the phenotypes of a genetic peroxisomal disorder that is seldom managed by neurosurgeons. We report an unusual case of cerebral X-ALD presenting as an enlarging CVC in a child, and discuss its nuances in corroboration with the literature. A previously well six-year-old male presented with confusion and fever. Urgent neuroimaging demonstrated a large CVC with resultant hydrocephalus. Of note, there were symmetrical areas of signal changes in the periventricular white matter bilaterally involving the corpus callosum, thalami, cerebral peduncles, midbrain, and pons in his MRI. Further investigations performed as part of his medical workup reported high plasma concentrations of very long-chain fatty acids (VLCFA). Put together, a diagnosis of cerebral X-ALD was confirmed. Initially, an external ventricular drain was inserted directly into the CVC under stereotaxy to decompress it. Subsequently, endoscopic fenestration of the CVC was performed as the definitive treatment. He recovered uneventfully from the neurosurgical interventions and proceeded for the treatment of his cerebral X-ALD. To our knowledge, this is the first report of cerebral X-ALD presenting as a CVC in a patient. This case adds to the limited literature for both rare conditions and highlights the importance of a multidisciplinary approach to management.

## Introduction

A cavum vergae cyst (CVC) is a rare midline cerebral malformation bounded anteriorly by the columns of the fornices, superiorly and posteriorly by the splenium of the corpus callosum, and inferiorly by the commissure hippocampi [1]. Together with other midline intracranial cysts, such as cavum septum pellucidum and cavum velum interpositum cysts, the CVCs usually do not require neurosurgical intervention in patients who have them [2]. Nonetheless, there have been some reported cases of these non-communicating lesions causing neurological symptoms via the obstruction of the ventricular system [1,2]. Separately, X-linked adrenoleukodystrophy (X-ALD) refers to a spectrum of rare, genetically determined metabolic spectrum disorders caused by excessive accumulation of very long-chain fatty acid (VLCFA) in tissues and plasma [3]. This is a condition seldom managed by neurosurgeons. Common clinical manifestations include dysfunctions of the central nervous system (CNS), adrenal glands, and testicles [3,4]. Here, the most severe clinical phenotype is the childhood cerebral X-ALD [5]. Early diagnosis and treatment are critical to alter the disease outcome before irreversible neurologic disability occurs [3]. We report an unusual early presentation of this condition in a child and highlight management nuances via a multidisciplinary approach.

## Case presentation

**Figure 1:**
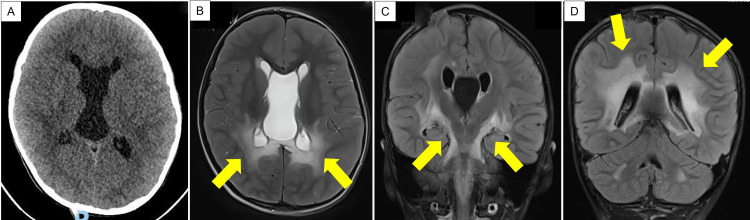
Representative neuroimaging of the patient at the time of initial presentation (A) Representative non-contrasted CT axial image depicting enlarged cavum septum pellucidum et cavum vergi with hydrocephalus. (B) Representative MRI axial image delineating a cavum vergae cyst causing hydrocephalus in T2-weighted sequence. Symmetrical areas of T2-weighted signal are noted in the periventricular white matter of bilateral parieto-occipital regions (yellow arrows). These latter findings were initially interpreted as transependymal CSF seepage due to obstructive hydrocephalus. (C and D) Representative MRI images in FLAIR sequence in coronal views. Both images highlight the extent of the signal changes in the periventricular white matter (D), especially involving lateral aspects of both thalami, cerebral peduncles, midbrain, and pons (C) (yellow arrows point to pertinent areas of MRI signal changes in both images). FLAIR: fluid-attenuated inversion recovery

A previously well six-year-old male with no significant past medical or family history presented with a three-day history of worsening altered sensorium associated with fever. Of interest, it was noted that he had generalized skin hyperpigmentation involving his face, trunk, limbs, and mucous membranes. This finding was not observed in his immediate family members. Initial blood investigations showed no evidence of infection, but his serum sodium was low (126 mmol/ L). An urgent non-contrasted, computed tomographic (CT) scan of his brain initially reported a large cavum septum pellucidum et cavum vergi with hydrocephalus. Further delineation with a follow-up magnetic resonance imaging (MRI) brain demonstrated there was an enlarged cyst of the cavum vergi measuring up to 3.2 cm in diameter. This structure was interpreted as causing obstructive hydrocephalus with transependymal seepage of cerebrospinal fluid (CSF) (Figure 1).

The patient was referred to the neurosurgical team who inserted an external ventricular drain (EVD) under stereotaxy into the enlarged cavum vergi cyst to relieve the raised intracranial pressure. Intraoperatively, the cyst fluid was observed to be under high pressure and turbid. At this point in time, the working diagnosis was that of symptomatic hydrocephalus secondary to meningoencephalitis. As part of his management, a multidisciplinary team was coordinated to ascertain the underlying cause of his clinical presentation.

Postoperatively, the patient underwent extensive investigations as part of his diagnostic workup. Further tests demonstrated that the hyponatremia was secondary to the syndrome of inappropriate antidiuretic hormone secretion (SIADH). Fluid from the CVC had a high protein level (11 g/L) but was otherwise negative for any infective or autoimmune markers. The genetics team was consulted to assess his unusual pattern of skin hyperpigmentation. A failed short Synacthen test confirmed primary adrenal insufficiency. The workup for peroxisomal disorders showed that his very long-chain fatty acid (VLCFA) analysis had a high concentration of hexacosanoic acid (C26) with concurrent abnormal ratios of other VLCFAs (C26: C22 and C24: C22). This profile was consistent with a diagnosis of X-ALD. Next, a closer review of the preoperative MRI brain images noted there were symmetrical areas of increased T2-weighted/fluid-attenuated inversion recovery (FLAIR) signal changes in the periventricular white matter of bilateral parieto-occipital regions, involving the corpus callosum, thalami, cerebral peduncles, midbrain, and pons. Based on the neuroimaging criteria, his Loes score was 9-10 [6]. Put together, the patient was likely to have a concurrent diagnosis of cerebral X-ALD. (Table 1).

**Table 1:** Summary of relevant blood and CSF investigations performed as part of the workup

INVESTIGATION	RESULT	REFERENCE RANGE
Hemoglobin	11.9	11.4 to 14.2 g/DL
Hematocrit	32.2 ¯	32.8 to 41.1%
White blood cell count	12.08	5.22 to 13.35 10(9)/L
Neutrophil absolute	8.72 ­	1.63 to 7.01 10(9)/L
Lymphocyte absolute	2.36	1.93 to 7.25 10(9)/L
Eosinophil absolute	0.02	0.00 to 0.84 10(9)/L
Platelet count	289	140 to 440 10(9)/L
C-reactive protein	6.9	0.0 to 5.0 mg/L
Procalcitonin	1.28	≤ 0.09 µg/L
Sodium, serum	126 ¯	138 to 145 mmol/L
Potassium, serum	4.2	3.4 to 4.7 mmol/L
Bicarbonate, serum	20	14 to 23 mmol/L
Chloride, serum	93 ¯	98 to 107 mmol/L
Urea, serum	3.1	3.2 to 7.9 mmol/L
Creatinine, serum	36	27 to 54 µmol/L
Calcium (adjusted by Albumin)	2.24 ¯	2.30 to 2.63 mmol/L
Total Calcium	2.12 ¯	2.30 to 2.63 mmol/L
Magnesium	0.64 ¯	0.86 to 1.17 mmol/L
Alanine transaminase, serum	28 ­	9 to 25 U/L
Protein Total, serum	61 ¯	64 to 77 g/L
Bilirubin Direct, serum	7 ­	1 to 3 µmol/L
Aspartate Transaminase, serum	63 ­	21 to 44 U/L
Alkaline Phosphatase, serum	144 ¯	166 to 393 U/L
Albumin, serum	34 ¯	35 to 45 g/L
Gamma-Glutamyl Transferase, serum	12	6 to 15 U/L
Bilirubin Total, serum	16	3 to 21 µmol/L
Osmolality, serum	260 ¯	275 to 300 mOsm/kg
Osmolality, urine	764	50 to 1200 mOsm/kg
Sodium, urine	172 mmol/L	Not applicable
Thyroid stimulating hormone, serum	0.50	0.50 to 4.5 mIU/L
Thyroxine (T4) free, serum	13.3	10.3 to 25.7 pmol/L
Short Synacthen test	106 nmol/L à 107 nmol L à 102 nmol/L	
Docosanoic acid (C22)	28 ¯	30 to 112 µmol/L
Tetracosanoic acid (C24)	52	14 to 80 µmol/L
Hexacosanoic acid (C26)	1.89 ­	0.33 to 1.50 µmol/L
C24/C22 ratio	1.86 ­	0.44 to 1.05
C26/C22 ratio	0.068 ­	0.005 to 0.030
Phytanic acid	1.06	0.20 to 19.30 µmol/L
Pristanic acid	0.07	0.00 to 2.00 µmol/L
Red blood cells, Cyst fluid	6 ­	≤ 0/µL
White cell count, Cyst fluid	1	0 to 5/µL
Glucose, Cyst fluid	3	2.4 to 4.3 mmol/L
Protein total, Cyst fluid	11 ­	0.10 to 0.40 g/L
Microscopy, Cyst fluid	No organism seen	Not applicable
Culture, Cyst fluid	No bacterial growth (3 days)	Not applicable
Autoimmune encephalitis panel, Cyst fluid	No abnormality detected	Not applicable

**Figure 2:**
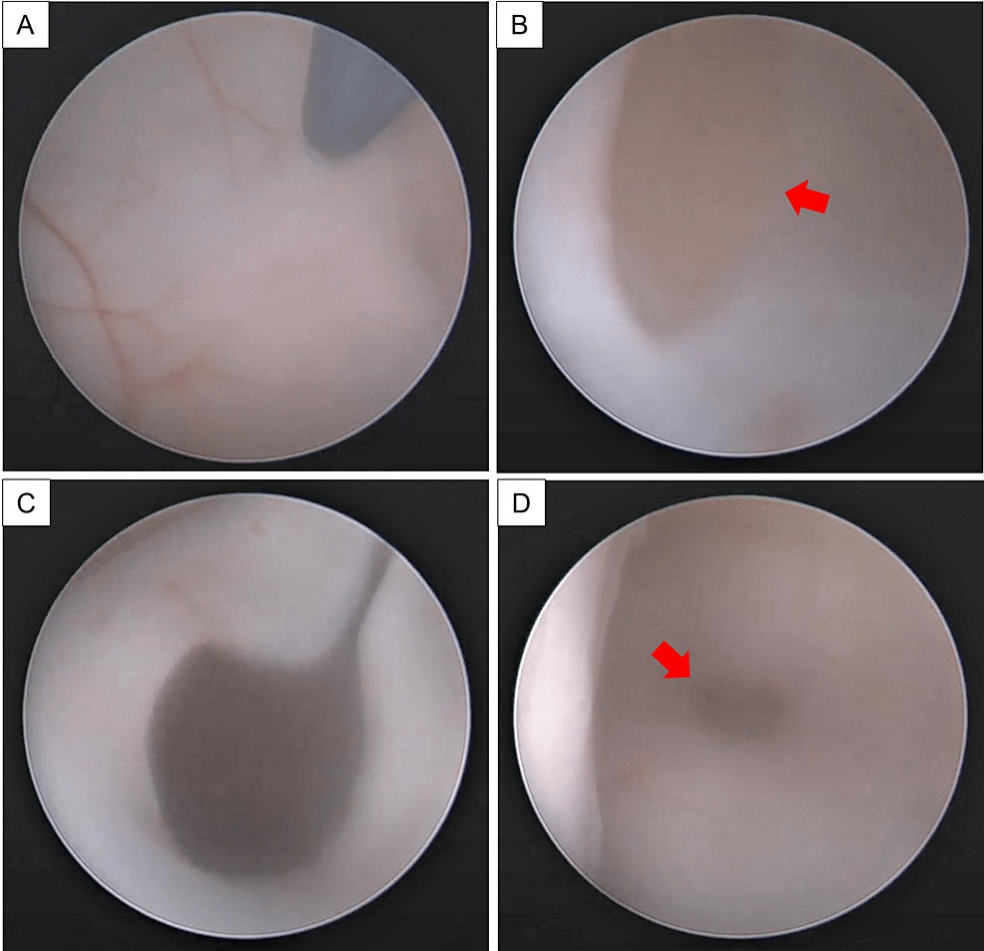
Intraoperative photos taken during the neuroendoscopic procedure for CVC fenestration (A) Septostomy with the blunt end of a monopolar probe; (B) Yellowish cyst wall visualized under the septostomy (red arrow); (C) Post-fenestration of CVC with collapsed septostomy walls and (D) Stoma on the contralateral side (red arrow).

With regard to his hydrocephalus, the decision was made for an endoscopic CVC fenestration and septostomy to communicate with the ventricular system. Briefly, a 0° wide-angle rigid neuroendoscope with two working channels (Little LOTTA, Karl-Storz, Germany) was inserted into the existing burrhole. Blunt perforation was performed via a monopolar coagulation probe. The CVC was then fenestrated until it collapsed, allowing the contralateral ventricular wall to be visualized. Excess bleeding from the cyst wall was addressed with continuous irrigation or coagulation [7]. This was performed successfully, and the existing EVD was removed at the end of the procedure (Figure 2).

The patient recovered to his baseline neurological status and commenced treatment for his newly diagnosed cerebral X-ALD. Approximately three months later, the patient was readmitted for concerns of raised intracranial pressure. An MRI brain reported a recurrence of his CVC and hydrocephalus. He underwent a repeat endoscopic re-fenestration of the CVC. Intraoperative findings confirmed the previous stoma had closed and the recurred CVC was obstructing the foramen of Monro. As before, the patient’s postoperative recovery was uneventful. Subsequent neuroimaging during the course of his follow-up showed the CVC remained reduced in size without evidence of hydrocephalus.

## Discussion

The cavum vergae is the posterior extension of the cavum septum pellucidum and is a persistence of the embryological fluid-filled space between leaflets of the septum pellucidum [8,9]. This congenital anomaly is estimated to be present in up to 30% of newborns and persists in 1% of adults [10]. Although the cavum vergae often coexists with the cavum septum pellucidum (also known as ‘cavum septum pellucidum et vergae’), there have been previous observations of the cavum vergae occurring independently [11]. When these midline cysts expand, the foramina of Monro and the third ventricle are obliterated, resulting in life-threatening sequelae of obstructive hydrocephalus [9]. Under such circumstances, urgent neurosurgical intervention is necessary to restore CSF equilibrium. Common approaches described in the literature include endoscopic fenestration, shunting, open surgery, and stereotactic fenestration [1,12,13]. A recent systematic review reports that surgical treatment provides a resolution of symptoms for most patients, regardless of the choice of procedure [12]. These findings are consistent with our patient, who was initially treated with a stereotactic-guided external ventricular drain directly into the cyst, and subsequently required two interval attempts at endoscopic fenestration to manage his hydrocephalus.

Separately, childhood cerebral adrenoleukodystrophy is the most devastating phenotype in X-ALD patients [5,14]. Typically, affected patients have normal development until they reach four to 10 years of age, at which time behavioral changes surface, typically as attention deficit hyperactivity disorder (ADHD). In selected cases, they may also have hyperpigmentation of their skin and mucous membranes [15,16]. As the disease progresses, deterioration of vision, hearing, and motor functions is observed [14]. In addition to CNS symptoms, adrenal dysfunction or gonadal insufficiency can surface. Generally. the diagnosis is established by the characteristic pattern of cerebral demyelination observed in neuroimaging, and simultaneous confirmation by biochemical measurement of plasma total lipid VLCFA [17]. Most commonly, the cause of VLCFA accumulation is due to a mutation of the ABCD1 gene-encoded protein adrenoleukodystrophy protein (ALDP) [18]. However, the exact mechanism of excessive VLCFA in causing abnormalities in the CNS and adrenal glands is not fully elucidated [5]. Pathological changes in the CNS are often characterized by a breakdown of myelin with relative sparing of the axons, accumulation of cholesterol ester, and perivascular inflammatory response with a breakdown of the blood-brain barrier [5]. To date, VLFCA levels are the main biochemical markers to diagnose peroxisomal disorders such as X-ALD [17,19]. In congruency with most clinical laboratories, we rely on the analysis of VLCFA levels in plasma as the gold standard [20]. A recent study has reported the use of C26:0-lysophosphatidylcholine (C26:0-LPC) levels measured in dried blood spots using liquid chromatography-tandem mass spectrometry and its superior diagnostic performance in comparison to traditional VLCFA analysis (that is, C26:0 and C26:0/C22:0 ratio) [20]. Although this is certainly a more intuitive clinical test to look forward to, it is yet to be implemented at our institution.

Adrenoleukodystrophy has the propensity to spread longitudinally along white matter tracts [6]. This results in a characteristic prolongation of T1-weighted and T2-weighted relaxation times seen on MRI scans [6,21]. To determine disease severity, the Loes score is applied. This is a semiquantitative 34-point scale based on the extent of MRI changes in different brain regions [6]. Clinically, the rapid neurological decline is caused by a severe inflammatory demyelination process primarily affecting the cerebral hemispheres. Studies report that up to 80% of patients initially show localized demyelinating changes in the splenium of the corpus callosum and subsequently progress to involve the adjacent parieto-occipital white matter. Alternatively, the initial demyelinating lesions may occur in the genu of the corpus callosum and then spread symmetrically or asymmetrically to the white matter of the frontal lobes [14]. In cases where brain neuroinflammation is detected early, disease progression can be arrested by hematopoietic stem cell transplantation [22]. In cases where no appropriate donor is available, autologous hematopoietic stem cell gene therapy may be considered in some centers [23]. To date, there is no previous report in the literature whereby cerebral X-ALD presents in a similar fashion as our patient.

Put together, it is unknown at this stage if there is a direct association between our patient’s CVC and X-ALD. Interestingly, separate studies on fluid from intracranial cysts and the CSF of cerebral X-ALD patients have been independently reported to have high protein levels [24-26]. Under such circumstances, we are uncertain if the increased protein demonstrated in our patient’s CVC is due to either pathology or both at this stage. We theorize that he had a pre-existing, asymptomatic CVC. A later onset of cerebral X-ALD may have caused demyelinating intraparenchymal changes and accumulation of neuroinflammatory products within the CVC. Consequently, the CVC enlarged with obstruction of the ventricular system. This then led to issues of raised intracranial pressure, further contributing to a central cause of SIADH at presentation. Nonetheless, we acknowledge this hypothesis is merely based on our observations and requires more scientific validation. Owing to the vastly different diagnoses for this patient, the emphasis is on open lines of communication and working closely between different subspecialties so that intervention and holistic care can be coordinated promptly.

## Conclusions

The authors therein report a unique case of cerebral X-ALD presenting as obstructive hydrocephalus from a CVC. In addition, we reinstate the need for continued in-depth research for better disease understanding for affected patients. Overall, this case adds to the limited literature for both rare conditions and highlights the importance of a multi-disciplinary approach to management.
